# Hydrogen

**DOI:** 10.3390/ma4122073

**Published:** 2011-11-30

**Authors:** John O’M. Bockris

**Affiliations:** Retired Distinguished Professor (1978–1997), Texas A&M University, 10515 SW 55th Place, Gainesville, FL 32608, USA; E-Mail: jbockris@cox.net; Tel.: +1-352-335-3843; Fax: +1-352-335-2925

**Keywords:** hydrogen, light, photoelectrocatalysis, corrosion, solar power, titanium-dioxide

## Abstract

The idea of a “Hydrogen Economy” is that carbon containing fuels should be replaced by hydrogen, thus eliminating air pollution and growth of CO_2_ in the atmosphere. However, storage of a gas, its transport and reconversion to electricity doubles the cost of H_2_ from the electrolyzer. Methanol made with CO_2_ from the atmosphere is a zero carbon fuel created from inexhaustible components from the atmosphere. Extensive work on the splitting of water by bacteria shows that if wastes are used as the origin of feed for certain bacteria, the cost for hydrogen becomes lower than any yet known. The first creation of hydrogen and electricity from light was carried out in 1976 by Ohashi *et al.* at Flinders University in Australia. Improvements in knowledge of the structure of the semiconductor-solution system used in a solar breakdown of water has led to the discovery of surface states which take part in giving rise to hydrogen (Khan). Photoelectrocatalysis made a ten times increase in the efficiency of the photo production of hydrogen from water. The use of two electrode cells; p and n semiconductors respectively, was first introduced by Uosaki in 1978. Most photoanodes decompose during the photoelectrolysis. To avoid this, it has been necessary to create a transparent shield between the semiconductor and its electronic properties and the solution. In this way, 8.5% at 25 °C and 9.5% at 50 °C has been reached in the photo dissociation of water (GaP and InAs) by Kainthla and Barbara Zeleney in 1989. A large consortium has been funded by the US government at the California Institute of Technology under the direction of Nathan Lewis. The decomposition of water by light is the main aim of this group. Whether light will be the origin of the post fossil fuel supply of energy may be questionable, but the maximum program in this direction is likely to come from Cal. Tech.

## 1. Introduction

Copernicus (1473–1543) discovered the heliocentric nature of our position in the universe. Newton (1643–1727), with his discovery of the inverse square law, was able to follow with equations which rationalized our position in a near sphere whilst we rotate around the sun. Planck (1858–1947) introduced us to the theory of quanta in energy physics. Freud (1856–1939) made us realize that most of what we are is unconscious inside us.

Of these grand founders of the world which we have made in four centuries, leaves out the extraordinary experimenter who discovered electromagnetism, Michael Faraday (1791–1867). If we tried to put in order of importance his discoveries, it would be difficult to find something more far reaching than electromagnetism. It led to our electric motors and our ability to communicate across the world. In fact, it has created our world more than any one of these great achievements after that of Newton.

It is not difficult to see the number two discovery of Faraday after electromagnetism [1]. He was the first man who found out how to split water, finding that this liquid consists of a couple of gases! One is oxygen upon which we directly depend. The other is Hydrogen!

**Figure 1:**
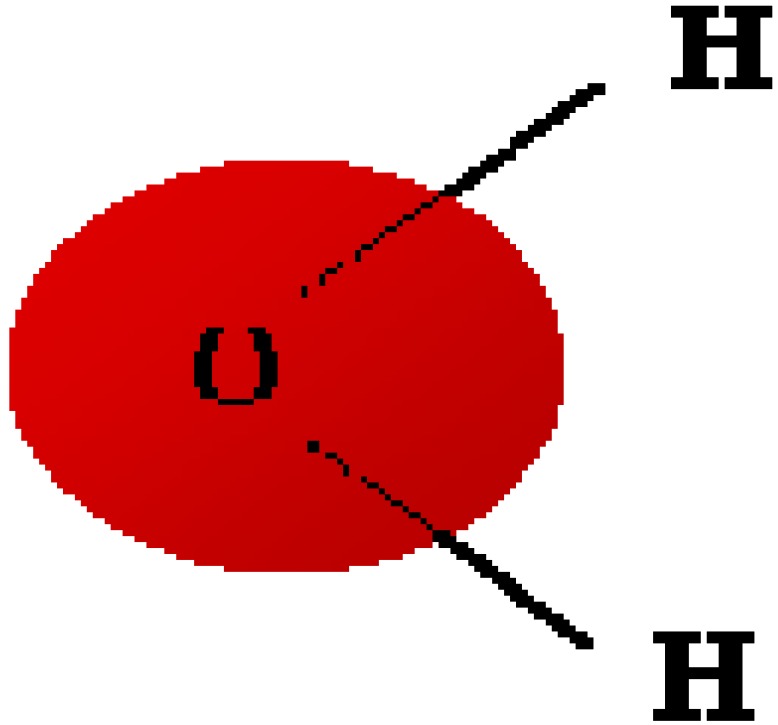
Water.

I have made this historical introduction to hydrogen because many do not yet realize the mega importance of hydrogen in our world. Our principal liquid is made up of two atoms of hydrogen and one atom of oxygen (see Figure 1).

Of what do we consist? Fats, proteins and water largely. What do these consist of? A good deal of it is hydrogen.

Finally, to stretch very far out, it is the essential of the hydrogen atomic bomb.

I understand the readers of this article may wish to hear more about light and hydrogen than the other aspects of our subject. So I am going to devote about half of the article to light and hydrogen and the rest to general aspects of this important element. I will bring the reader from 1980 to 2011 in respect to the light oriented aspects of the work.

## 2. Results and Discussion

### 2.1. The Electrical Synthesis of Hydrogen

What Faraday did when he split water was to make up what is now called a “cell”. There are two strips of metal: one drives out electrons to neutralize hydrated protons in the solution; and the other takes in electrons from OH^−^ ions if in the solution. The circuit is completed via a battery (invented by Volta in Italy in 1800). At the negative electrode (Faraday called this a cathode):

2H^+^ + 2e ➔ 2H_adsorbed on metal_

2H_adsorbed_ ➔ H_2gas_

This first sequence of reactions called the “Tafel Mechanism” is named after Julius Tafel [2], a chemist who was poor in health and had a lot of time to think. It was Tafel whose name sticks to an empirical equation by which he represented the results of electrolyzing water (1903).

η = a − b logi(1)

In this equation, “i” is the electric current density flowing through the cell. The current density is discussed in terms of amps per cm^2^ or sometimes milliamps per cm^2^.

The letters “a” and “b” Tafel found were constants, characteristic of the electrode. When the overpotential η is plotted against logi, the result is called a “Tafel line”. Overpotential is the extra potential which one has to add to the reversible electrode potential to make the reaction go in a given direction at a specific rate, represented by “i”, the current in amps divided by the size of the electrode in square centimeters.

Other steps or reactions are observed with other electrode materials. The reaction: H^+^ + e ➔ H_ads_ was singled out by Max Volmer and Erdey Gruz [3] in 1930 and called the “discharge step.” When the coverage θ of an electrode surface gets too high, the H^+^ discharges onto adsorbed H_ads_

H^+^ + H_ads_ + e ➔ H_2_

The first people to publish this last equation were Kobosew, and Nekrassow also in 1930 [4].

**Figure 2:**
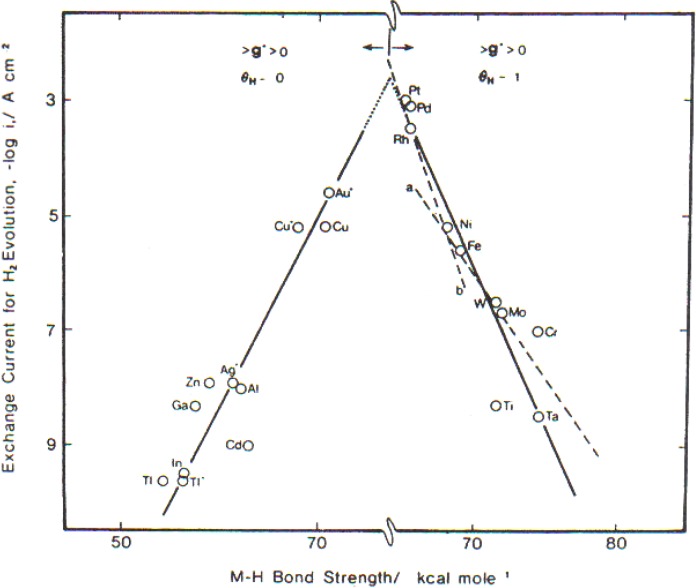
A plot of exchange current density for hydrogen evolution reaction *vs.* M–H bonding strength (Reprint from [5] and [6]).

When η = 0, the electrode is in a “reversible condition.” Its reactions go in both directions at the same rate. One can tell whether the Volmer step is rate determining because then the reaction goes faster when the M-H bond strength gets stronger (e.g., Hg, PG, Tl, *etc*.), but it goes slower on transition metals as the MH bond gets stronger (Ni, Mo and Ta). The metals make a sort of volcano (see Figure 2).

### 2.2. A Hydrogen Economy (1971)

By 1969, people who lived in Los Angeles and San Francisco were being bothered by unpleasant smog. It was identified as arising from reactions between compounds which come out of cars exhaust pipes. It was feared that smog would continue to spread over all towns which had sufficient automotive traffic (It was realized later on that the trouble of smog in Los Angeles was due to the absence of winds from the east along with a screen of mountains so the winds are not able to blow out the smog).

Of the possible solutions to the smog, one stood out as having far reaching benefits worldwide. It was the suggestion to replace gasoline with hydrogen on a grand scale [7]. Obviously, the positive side of hydrogen was that there would be no more trouble with smog from car exhausts anywhere.

In the next twenty years, as the automotive and oil companies controlled the contents of their fuel so that the smog abated, the realization of an even worse result of fossil fuels came to the fore—warming of the atmosphere due to CO_2_ absorbing light reflected from the earth [8]. Here again, hydrogen was suggested to replace gasoline in general because of the danger of global warming [9].

All seemed set for a Hydrogen Economy. However, the financially powerful oil companies did not want their business taken from them when they had plenty of oil still to sell even if they had to distill it from the plentiful tar sands. A Hydrogen Economy would not be in the oil companies’ best interests.

Some countries, notably Japan [10], have taken up the development of a Hydrogen Economy. As it nears actualization, some problems have arisen. The cost of hydrogen directly from the electrolyzer being driven by wind energy (or any one of the clean renewable energies like wind) could be low enough, but money would be needed for storing at a high pressure. To avoid embrittlement of pipes as a result of long term contact with hydrogen, special pipes made of nickel-steel would be required. Finally, one has to suffer a 50% loss of the energy to get the hydrogen back into electricity, the most useful form [7].

At 2011, there is still enthusiasm for hydrogen among those who work on our future energy supply. If we go on allowing fossil fuels and the CO_2_ to increase in the atmosphere, the temperature will eventually become too warm for us to live on this planet. A Hydrogen Economy [10] would end global warming.

Those who oppose the oil companies’ obvious wish to continue their business, have a weapon they can use: a carbon tax. Europeans are paying about twice the price for gasoline than do Americans. The thought is to bring up the price here to what Europeans are paying, over a 5 year period. The increased tax would help the government introduce an alternative fuel in place of gasoline.

Suppose a carbon tax [11] makes hydrogen cheaper to buy than gasoline? Then there comes another problem. Most people would prefer a liquid to fuel their cars. To go to a pressurized gas tank is not a pleasant thought for Americans who have been used to filing up with a liquid.

However, this particularly aspect can be solved and global warming can still become of no consequence (See next chapter).

### 2.3. “Liquid” Hydrogen

Hydrogen as a general base to our energy economy is certainly attractive. Many leading researchers in this area, for example, Turner of NREL [12] and Lewis [13] of Caltech, are in favor of Hydrogen. They would like to see solar energy developed to provide the energy for water to provide hydrogen.

Meanwhile, George Olah [14] of the University of Southern California has encouraged methanol as a better alternative fuel to gasoline than hydrogen. He regards methanol as a kind of liquid hydrogen. It has the good properties that hydrogen has but is easier to handle.

The methanol that Olah supports would have to come from renewables, otherwise it will introduce CO_2_ by the backdoor. Any of the renewables will do depending upon the local climate: e.g., in Australia-solar; and in northern climes-wind. Eventually enhanced geothermal energy can contribute perhaps widely.

Clean hydrogen from water electrolysis could be combined with CO_2_ to make methanol. However, getting CO_2_ from the atmosphere is not a light hearted matter because the CO_2_ concentration in air is only 0.03%. One alternate idea is to use biomass as suggested by Keith from the University of Calgary [15]. Biomass after all does come from the atmosphere according to the general equation:

$$CO_{2}+H_{2}O\overset{hy}{\to }(“CH_{2}O”)+O_{2}$$

“CH_2_O” indicate that this would be a building element of the polymers which make up our green plants. Obtaining it from green plants is simply a matter of combustion and separating out the CO_2_. It is clear that the CO_2_ that could be obtained from biomass would have indeed come from the atmosphere-perhaps with a delay of some decades, but nevertheless, it is bona fide atmospheric CO_2_.

It is easy to comprehend that if clean energy from renewables were used to form hydrogen and the CO_2_ was obtained from biomass, then the following reaction would be carried out:

3H_2_ + CO_2AT_ ➔ CH_3_OH_At_ + H_2_O

Methanol_AT_ made in this way would be a CO_2_ neutral fuel because when burned as a fuel the CO_2_ which is put back into the atmosphere was obtained from the atmosphere (through biomass).

For the version stated, I remain an advocate of hydrogen as a clean fuel. However, because of cost problems in handling it after production, I think it is better to shift over to methanol_AT_-methanol made with CO_2_ from the atmosphere.

There may be some misunderstanding here because I am claiming that it is possible to have a cheaper methanol than hydrogen and yet I am using three moles of hydrogen to form one of methanol. How is that possible? Well, I don’t say that the cost of methanol is a lesser cost than molecular hydrogen gas from the electrolyzer. I maintain that everything that is needed to use hydrogen (storage, transport and conversion) makes the final cost of using hydrogen in practice more expensive than methanol_AT_ for which you have to do none of these things.

CO_2_ directly from the combustion of a fossil fuel (e.g., CO_2_ from an IC engine) would give CO_2_ to the atmosphere. Reading about the way oil has been formed over the past billion years or so, its connection with being formed from the atmosphere is pretty remote. It is formed under high pressure under the earth and at high temperature [16,17].

### 2.4. Could Hydrogen at Low Cost Come from Bacteria?

The idea of bacteria producing hydrogen seems irrational; however, there is a large amount of work in the literature which attests to the fact that it is possible that there are “certain” bacteria that will produce hydrogen from water.

Nigel Packham [18] has become well known partly because he was the first person to produce tritium from deuterium (a nuclear reaction in the cold), and also due to his parallel work on obtaining hydrogen from bacteria (his thesis). He found small amounts of hydrogen from bacteria in about one year’s work. However, this work did not provide a line of bacteria that were particularly good hydrogen producers. This came later [19].

Jumping from 1990 to 2008, the work of an Indian scientist Debaratna Das comes to the fore. He discovered that INTEROBACTER CLOACAE DM11 has a relatively powerful hydrogen producing characteristic. Das discovered something even more intriguing, from the commercial point of view. He found these bacteria would live and work for some time if fed sewage sludge. This sludge comes from the last stage in normal treatment of sewage which produces a solid material usually burned or used on farms. This sewage sludge as feed for the bacteria made the cost very small: about $2.25 per 10^6^ BTU [20].

One MBTU is approximately the same in energy as one gigajoule. Thus, Das’ calculation could be compared with the cost of hydrogen by the more normal means such as wind energy driven electrolysis. These other costs are between $10–$20 per GJ. If we take the medium, $15 per GJ, and compare it with Das’ figure, then his is markedly lower. The only reason why it is not a matter of great rejoicing is the fact that it depends on the acceptability of sewage sludge in which it found the Interobacter bacterium. This makes one pause and ask whether there is some substitute for sewage sludge which might provide the same final product of hydrogen massively at less than that of the cost of other means. In fact, there has been found bacteria in cow dung that give rise to hydrogen easily which would provide a lost cost [21].

So let us leave it there for now but remember to consider seriously the bacterial production of hydrogen from bacteria found in certain wastes.

### 2.5. The Development of a Hydrogen Economy at Texas A&M University

The Hydrogen Economy began in 1971. I have written elsewhere about the meeting at General Motors in which a brain storming session gave rise to 3 out of 5 ideas of energy in the future which featured Hydrogen. The first full paper (Bockris and Appleby) on this subject was called, “The Hydrogen Economy”, published in 1972 [22].

I continued to play a part after the Hydrogen meeting in Miami organized in 1974 (900 attendees). The difference in respect to the contributions that Veziroglu and I made to the Hydrogen Economy is that after 1974, Veziroglu made the development of the new culture his entire scientific life from that time onwards until and after retirement. I continued to research hydrogen though, in many ways.

In the meantime, my earlier contributions to this matter were not unnoticed. The National Science Foundation turned to me when they wanted to set up a hydrogen-industry cooperation group. The idea here was that a University professor should be chosen to lead the research, but only half of the money would be given us directly from NSF for five years. In order to fulfill the conditions of the contract, it was necessary that I raise the other half of the money from industrial companies [23].

In 1982, Bill Craven was the business manager in my research group at Chemistry Department, Texas A&M University. He was an impressive person when it came to organization, contracts, money and management. He confidently convinced NSF sponsors that their money would be well handled.

In 1991, Veziroglu and I published a simple book called “Solar-Hydrogen”. It was reprinted in eleven countries [24].

So far in my brief account, I have left out one of the most important players—Hampton Robinson. Hampton Robinson was more important, from our point of view, as being a wealthy man.

We learned how to get Robinson to fulfill his promise that we should not need to concern ourselves with the government or the University for financial support. No list of needs seemed to activate him. He had to have his emotions stirred, which was easy because he thought a number of practices in the University were insufficiently supportive of its researchers. He was upset by the fact that big equipment costing, say, $100 k had to be sought by writing proposals, thereafter graded by our academic rivals, to government agencies. When Robinson found that we didn’t have what we needed to do our work, he emitted a polite expletive, “Disgraceful!” And one got used to seeing his hand go inside a pocket, drawing out his checkbook. Taking into account inflation at 4% per year, Robinson provided as much as $2 million in 2011 money [25].

We agreed that the major objective we would concentrate on would be light and hydrogen: the idea that we could take solar light and play it upon an electrochemical cell containing two electrodes, each one of which had a semiconductor as an electrode. From this meeting between the incoming light and the semiconductors, hydrogen would be evolved at the p type electrode and oxygen at the n type. There is more to say than this, but that was the essence of it and that is what we tried to do and had some success in doing.

### 2.6. Light Interactions with Water Carried out at Texas A&M University

As we began to dig into the state of the theory in 1982, I found that its concepts had been influenced by the noted German electrochemist Heinz Gerischer who at that time was the Director of the Max Planck Institute in physical chemistry. Basically, it is breaking through his concepts which were strictly Schottky Barrier and not different from what would have been the right equations to apply to an interface accepting light, but without taking into account the surface and its structure; *i.e.*, no hint of a double layer structure in the solution-semiconductor interface.

**Figure 3:**
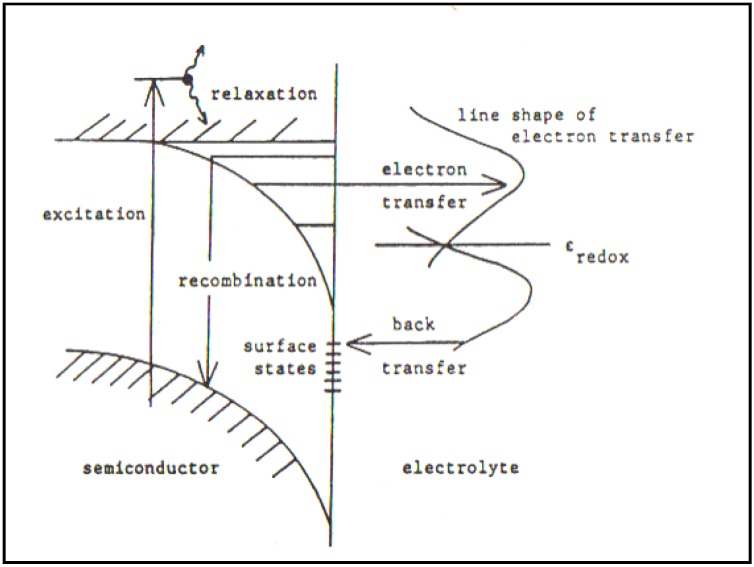
Electronic processes occurring at a semiconductor/electrolyte junction under illumination (Reprint from [28] and [29]).

**Figure 4:**
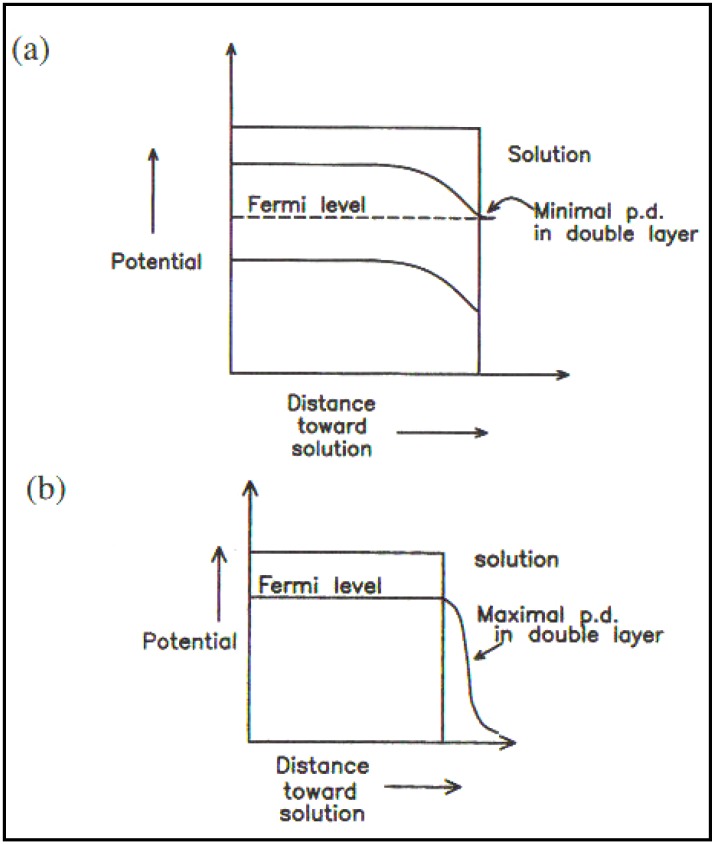
(**a**) The semiconductor/solution interface (no surface states); (**b**) The metal/solution interface (Reprint from [29]).

Uosaki and Kita were Japanese coworkers who had worked with me in Australia in the 1970s. They published a paper [26] which sought to show up the evidence that there were surface effects in Photoelectrochemistry so that the Gerischer oriented concepts would have to undergo a radical revision [25,26,27] (See Figure 3 and Figure 4).

Several theoretical initiatives are, at this section of the work, due largely to Khan who gave us tools with which we could work and deal with two extremes. On the one hand was the ideal model stressing the internal part of the semiconductor (similar to models used by Gerischer). Then there was the other extreme in which the internal part of the double layer, which we still have effects due to the potential gradient and concentration gradient which effected the electron movements would be coupled with influence of an electrical double layer and of surface quantum states [30].

**Figure 5:**
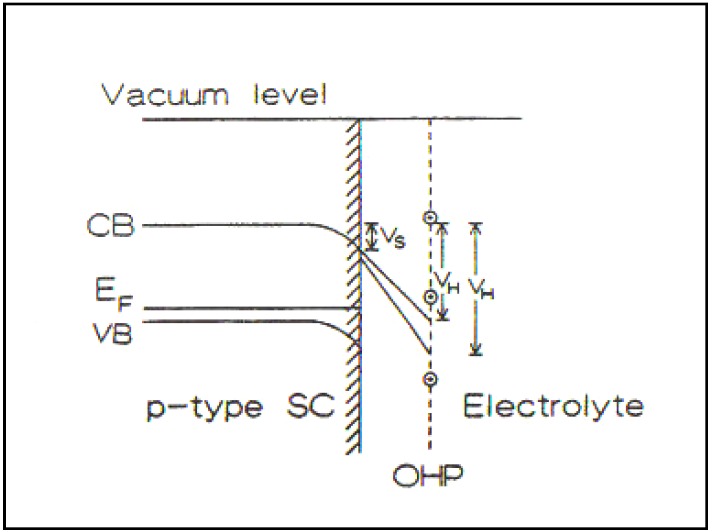
*p*-Type semiconductor/solution interface in the presence of high-density surface states. The potential difference in the Helmholtz part of the double layer, (*i.e.*, that in the solution) is greatly increased compared with a situation with a negligible number of surface states. Correspondingly, the potential difference within the semiconductor is greatly diminished compared with one containing negligible surface states (Reprint from [29]).

In Figure 5, there is a diagram which illustrates the effects of the more realistic models which we developed. They are meant to show that there are two short influences upon the electron, stimulated by the photons which the electrode absorbs: on the one hand for the internal forces, the gradients of concentration and a potential; but on the other hand, there was the electrical double layer highly developed for metals in its functioning at metal-solution interfaces.

### 2.7. Surface States

**Figure 6:**
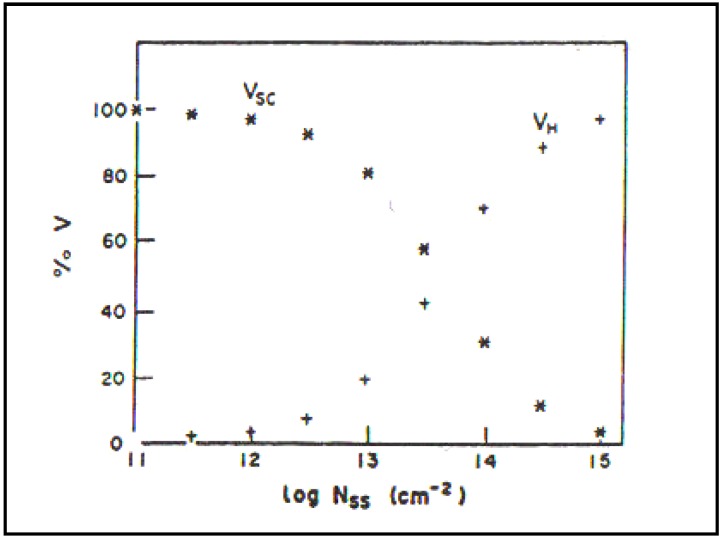
Relative potential drop in the space charge region and in the Helmholtz region as a function of surface state density (Reprint from [29] and [32]).

A change in thinking came from Khan’s theoretical work on the conversion of light to electricity and then to hydrogen [31]. This was accompanied by the evidence that the performance of p type semiconductors evolving hydrogen is highly surface dependent! In the former thinking, the rate determining step was always inside the semiconductor (Schottky Barrier). For us, this was not the whole story (Figure 6).

**Figure 7:**
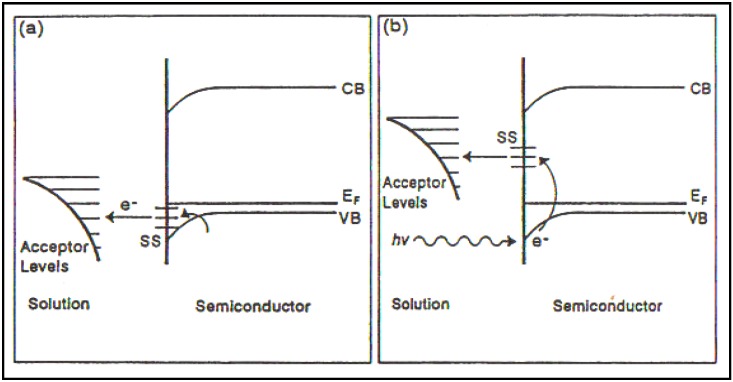
Representation of a “good” surface state on a semiconductor electrode. (**a**) Thermal activation and (**b**) photoexcitation of an electron from the valence band to surface states (Reprint from [29]).

Detecting and determining surface states became priority in our group and had to be tackled system by system (See Figure 7).

Calculations by Khan [33] show the presence of a significant concentration of surface states (more than 10^12^ cm^−2^) begins to move the p.d. of the surface outside the semiconductor (see Figure 7).

**Figure 8:**
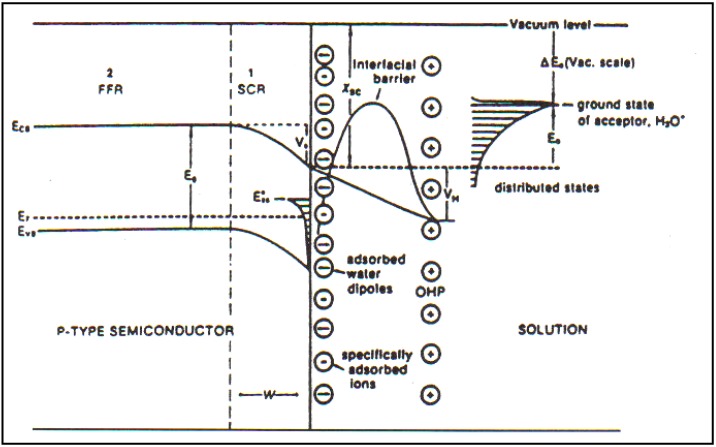
A schematic representation of a *p*-type semiconductor/solution interface (Reprint from [29] and [33]).

It is possible to have semiconductor-solution contacts which are Schotty, but the majority states to which the model interface as in Figure 8.

### 2.8. Photoelectrocatalysis

A metal catalyst is sprayed onto the semiconductor in low surface concentrations (for how it is introduced, see below).

Sklarzcyck and Contractor [34] were the first to find out that platinum and other metals had a catalytic effect so long as they were added to the surface in tiny mounds. The total amount of the surface covered should be less than one half. Some of the earlier users, mistaking how the method was to be used, simply plated platinum on the semiconductor whereupon it stopped the functioning as a semiconductor all together.

Now, here is the key to the catalysis. Without using this method of light spraying with at least half of the semiconductor free to contact the solution, the catalysis does not work.

The essence of effective photoelectrocatalysis is to deposit platinum on to the catalytic surface in groups each containing a few hundred atoms. It is essential to allow free access between solution and semiconductor for the most part. Less that one-half of the semi-conductive surface is to have any contact with the catalyst.

**Figure 9:**
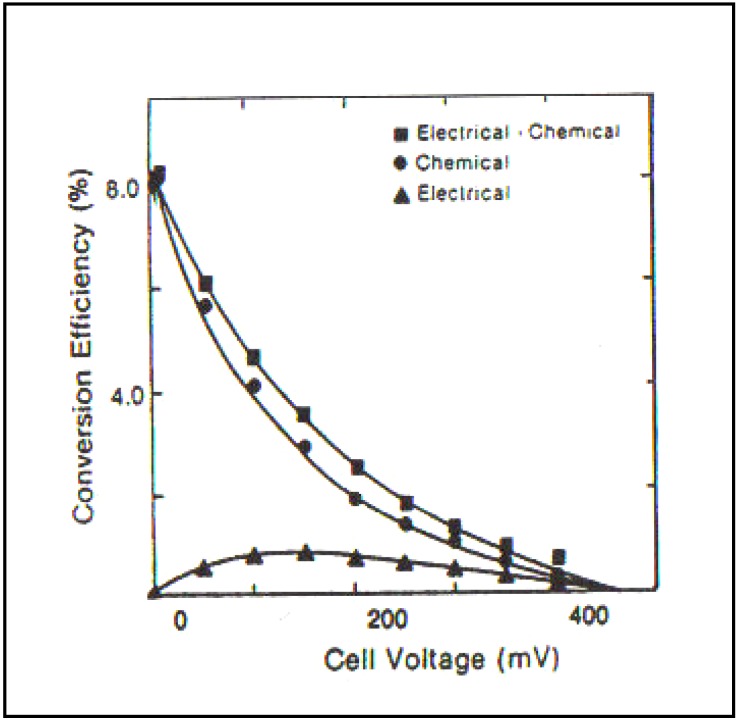
Chemical, electrical and total (chemical + electrical) efficiency of self-driven photoelectrochemical cell employing *p*-InP (Pt) and McO_2_-coated *n*-GaAas electrodes in 6 M KOH solution as a function of cell voltage (Reprint from [29] and [35]).

Photoelectrocatalysis is effective when performed as above. In the case of Sklarzcyck’s work and of the several coworkers who followed him, it gave rise to more then a 10 times increase in hydrogen evolution. Some efficiencies greater than 10% were achieved. It must be understood that these pioneer works were carried out in the 1980’s and were fully novel at that time (Figure 9).

Photoelectrocatalysis takes place with platinum but can be seen with several other metals [36,37].

What seems to be a good example of the effect of photoelectroncatalysis was found by Jeff Wass, a graduate student who was to be particularly effective in the photo-electrochemical reduction of CO_2_ [38].

### 2.9. The Splitting of Water by Light

It was realized in the 1980s that a mass production of hydrogen from water would be needed. The main methods being used to produce hydrogen at that time were connected with the steam reforming of natural gas (methane). At that point, few people realized the use of hydrogen as a fuel could eliminate CO_2_ and therefore global warming.

In 1980, I published a book called “Energy Options” [39]. This book has an entire chapter headed, “The Splitting of Water” so it was a novel objective 30 years ago. Among the methods which were seen as worth developing was heating water to 3,000 °K which would split it a little in order to get finally some hydrogen. Recombination of the dissociated water was a problem.

Then there were “cycles”. The protagonists got a number of reactions, some having positive entropies and some having negative entropies to be used at different temperatures. The temperature which you would have to get the hydrogen was reduced or increased depending on the sign of the entropy change. However, there was the problem of completeness in these reactions so the method has since faded.

In 1980, electrolysis gave 100 ma cm^−2^ for the fuel cell pioneers Tom Bacon and Rex Watson. Bacon and Watson worked in Cambridge but not at the University—rather in an abandoned air field close to it. They reported 1.4 volts which was record low volts at the time. General Electric reported 1.3 volts but this was because they were electrolyzing steam.

The photo-splitting of water was first observed accidently by Fujishima and Honda in 1972. The efficiency was about 1%. Fujishima was kind enough to describe to me the birth of a big field. He was in his laboratory and happened to be standing at the tap spraying water to wash some TiO_2_ crystals. He noticed that there appeared to be bubbles being formed on the crystals and they did not look the same as the bubbles which were ricocheting off it due to the tap water being poured. He turned the tap off but the bubbles were still coming so he reported this to Honda and the conclusion was made that somehow light on TiO_2_ in the presence of water was producing hydrogen and oxygen. In days, these two workers had a cell with one electrode platinum and the other TiO_2_ and light directed on the TiO_2_ electrode-splitting water. I think this was a remarkable discovery.

The evolution to a Stand Alone photogeneration of hydrogen from water with electrodes receiving light took three steps after Fujishima and Honda:

Step 1. One had to evolve away from the Schotty barrier. I do not mean that the Schotty barrier disappeared, but it didn’t have the same role anymore because it was not the only thing that had to be considered according to the Schotty view. It was not until Uosaki and Kita published a paper where they drew attention to the fact that an electrode heavy with surface states could be a good electrode for producing hydrogen after light was placed upon it [26].
Step 2. The realization of photoelectrocatalysis (Szklarzcyck and Contractor) [33].
Step 3. The realization that photoanodes were possible although the theory showed that the best ones from the photoelectrochemical point of view would dissolve anodically when used and thus be unstable (protected by a photo inactive oxide). The solution to this was first brought to light by our team in the Chemistry Department at Texas A&M University [40]. Using these new results, it was possible for Kainthla, Barbara Zeleny and Bockris to publish a stand alone solar light driven cell with 9.5% in 1987 [41].

### 2.10. Work on Water Splitting at the National Renewable Energy Laboratory (D.O.E.)

John Turner at the National Renewable Energy Laboratory has produced notable and progressive work in the matter of producing hydrogen from water by means of light and has given a substantial survey of the clean energy needs in the United States [42,43,44]. He at first drew attention to the uselessness of sequestration of the wastes from the present method of energy production (producing CO_2_ and other problems).

John Turner’s recommendation for a clean energy source was wind [12]. He pointed out that Middle America is well supplied with wind belts in which the average wind is several miles per hour above the average wind in other places. This wind potentially could supply energy to much of Middle America.

Sea based wind farms would supply energy for the east and west parts of the countries. No problem with space. There is an increase in cost for wind turbines on the sea because of extra engineering for safety and cost of cable transport of electricity to share. However, this is compensated by the presence of higher winds at seas.

Furthermore, the pay back time for wind power is relatively short at 3 to 4 years [12]. The cost of the electricity from wind power can be as low as 0.03 cents per kWh. The Wind Association of America predicts that in a few years they could supply wind based electricity at a cost of two cents per kWh.

There are other schemes for collecting wind energy which have not yet been brought into practice but have received supporting evidence. I am referring to the work of Roberts *et al.* [45]. He and his group purchased a helicopter, equipped it with four rotors and flew it up to 15,000 feet. They were able to generate electricity and take it down to the ground on a conducting tether which held the helicopter in position. At 15,000 feet, the winds in wind belts are high (75 mph).

Turner points out that an easy availability of hydrogen would provide energy for transportation at 50% efficiency compared with the 25% efficiency which is the upper line of the efficiency for burning gasoline in running cars.

The question of storage of this energy comes up. There are schemes but my own contribution to this is that all the hydrogen needed would be supplied at gas stations. Gas stations have electricity. The electricity only needs to be converted to DC to electrolyze water to give the needed hydrogen. The hydrogen would be available as easily as one uses electricity [12]. No storage at high pressure or high cost pipes of nickel steels.

### 2.11. Work Done at Duquesne University: Use of the Reduction of the Energy Gap in TiO_2_, a Low Cost Photovoltaic

Researchers have been working on n-TiO_2_ for at least 40 years [46,47] due to its high stability but could not improve its photo conversion efficiency for water splitting. This was because n-TiO_2_, due to its high band gap, could only absorb uv radiation which is approximately 5% of the solar spectrum. Thus Khan *et al.* [48] pioneered with the discovery of visible light active, highly stable, carbon modified (CM)-nTiO_2_ which can photoelectrochemically split water to hydrogen and oxygen with a photoconversion efficiency of 8.85% under xenon lamp illumination.

Comparing xenon light on modified TiO_2_, this is a 10× increase in efficiency; *i.e.*, the TiO_2_ is 10× more than the unmodified polarity. It should be noted that carbon incorporation in the lattice of n-TiO_2_ helps to lower the band gap and make it more active to visible light even though actual sunlight efficiency might be nearer than that with the xenon light. The fact remains that the carbon modified TiO_2_ (carbon modified) is both highly stable and 10 × more active than the regular n-TiO_2_.

To be compared with the efficiency stated, one should look at the work of J.A. Turner [49] reported photo conversion efficiencies up to 12.4% for water splitting using n gallium idiom phosphide 2 as the photo electrode and Licht [50] reported 16.3% using AlGaAs/Si as the photoelectrode in their cell. However, both photoelectrodes used by Turner and by Licht were unstable once they were used in the electrolyzed solution.

The appropriate correction for the use of a helping battery was used by subtracting an appropriate potential from the one measured [48].

### 2.12. Work Being Carried out in the Consortium at the California Institute of Technology

A consortium of encouraging size has been gathered at the California Institute of Technology in Pasadena, California to investigate the optimal methods which would lead to supply energies after the fossil fuels are gone. The leader of this team is Nathan Lewis, a Caltech professor who is well known in the energy world.

It is appropriate to begin with a paper which is a comprehensive account of the theory of photovoltaics and its interaction with light for p type cathodes which can absorb photons to overcome their energy gap and to land them in the conductivity band whereupon they form an electric current some of which can reach the surface and the outer circuit.

Here, one comes to the first requirement of a good photo electrode. It must have an Eg low enough for some of its electrons to leap over the energy gap and land in the conductivity band (analogous conditions apply to n type photo anodes). It is a useful summary (CF Khan and Bockris) [51]. Lewis brings in the theory of electron transfer at metal solution interfaces one version of which is given by the Marcus Theory. However, the Marcus theory is inconsistent with a number of facts such as the wrong gradient for the Tafel line [52]. The transfer at metal solution interfaces obeys the Tafel law current exponentially dependent upon overpotential. The equation which Marcus has been publishing for many years does not correspond to this law.

In 2006, Prof. Lewis has been joined by Dr. Nocera of MIT to review the world need for energy [13]. They are correct in citing CO_2_ in the atmosphere as banning the continued use of any carbon containing fuel. Readers may not realize what these authors are advocating that we should stop using gasoline!

Nocera and Lewis [53] would be joined here by most scientists. What is not stressed in the review is the darker side of the sun which gives an average of only 5.5 hours of useful light per day on average and this means a need for a partner substance for storage for the 19 “dark” hours. Nocera and Lewis agree that hydrogen is the best of the candidates to receive the hydrogen as the interim medium in the dark hours. Crabtree and Lewis, in another review of the entire problem, show a useful table (Table 1) of the various efficiencies of solar light conversion.

**Table 1:** Solar energy conversion efficiencies [53].

	Laboratory Best *	Thermodynamic Limit
Single junction		31%
Silicon (crystalline)	25%	
Silicon (nanocrystalline)	10%	
Gallium arsenide	25%	
Dye sensitized	10%	
Organic	3%	
Multijunction	32%	66%
Concentrated sunlight (single junction)	28%	41%
Carrier multiplication		42%

*As verified by the NREL Organic cell efficiencies of up to 5% have been reported in the literature.

Viewing this table in 2007 through the eyes of one of my colleagues who works in a company making pv couples, the highest efficiency available for purchase of a practical size cell at that time was 16%. Certainly far higher efficiencies have been measured but only for small experimental cells in the laboratory. Directly one attempts to enlarge them towards a 24″ diameter, the cells lose maximum efficiency.

Cost is still the block for the massive use of high efficiency solar energy. The solar-hydrogen group at DOE cannot see a solution on the horizon. By the time Crabtree and Lewis [53] wrote their paper (2007), they did not know of Tester’s paper from MIT on the development of enhanced geothermal energy, which he and his group proclaim would be massively available at 0.039 dollars per kWh.

In a corresponding review of the world’s energy needs, Lewis presents some startling facts. The USA needs 3 terawatts, but what was more frightening is Lewis estimates that the rest of the world needs 7 terawatts [51].

In the end, Lewis comes to the conclusion: It is solar-hydrogen. His comprehensive review is not basically founded on the solution to global warming but this is an important part of its affect [54].

Then in 2008, there appeared a publication from Lewis’ group at Cal Tech which describes one of the darker side of photovoltaics because there are losses and the losses are analyzed here for silicon in detail. Impurities are still the problem [55]. Workers who actually are building the cells right now tell me that this gap between 32% and 16% arises because of the difficulty of controlling impurities in manufacturing bigger cells.

Some new work is published on nano science cells [52] and described from the raw material upwards to the super clean cell. Surprisingly, the very clean cells for a reason unclear to the reader have photo potentials half those of larger cells.

One of the new methods brought up by the Lewis group was published in 2009 and concerns an opportunity to which we had also contributed at Texas A&M University (Kainthla and Khan) [41]. One of the conclusions is that because of the logarhythmic dependence between the energy gap and photocurrent, a small difference in Eg means a relatively large a change in photocurrent.

One of the leading factors in stand alone photo cells is the anode [56] According to Contractor and Bockris *et al.* [47] only a small range of photovoltaic properties of anodes are acceptable from a purely photovoltaic point of view. Thus, many n type electrodes, when used as anodes in a photoelectrochemical cell, dissolve and are not acceptable.

Katz and Lewis tried to use KTaO_3_ (full details given about this cell and its dimensions in the publication) but the net result was on the good side–that the KTaO_3_ does not dissolve under anodic conditions but the photo response is poor [56,57].

Perhaps the most widely valuable paper of all those published in association with Lewis is a collection of properties which are needed for water splitting cells and all the elements which may be useful [58]. I wish I had this collection of facts which are embraced in this considerable paper when I was working under NSF support at Texas A&M University. This paper deals with a bending of bands, the effect of this phenomenon on purity levels on the surface and in improved efficiencies which can thus result.

## 4. Conclusions

The Lewis group has a large opportunity to contribute to the national problem of what is going to replace the fossil fuels. There is no doubt that solar has its part to play in specific parts of the world (Australia and not forgetting North Africa). With a Director of proven ability in Nathan Lewis, and the large federal support he has gathered, it is difficult to expect better progress in the Solar-Hydrogen Economy to be achieved other than in the large group under his direction. I thank discussions with S.U.M. Khan and Kainthla in composing this paper.
